# Evolutionary relationships among salivarius streptococci as inferred from multilocus phylogenies based on 16S rRNA-encoding, recA, secA, and secY gene sequences

**DOI:** 10.1186/1471-2180-9-232

**Published:** 2009-10-30

**Authors:** Jean-François Pombert, Viridiana Sistek, Maurice Boissinot, Michel Frenette

**Affiliations:** 1 Canadian Institute for Advanced Research, Evolutionary Biology Program, Department of Botany, University of British Columbia, Vancouver, BC, V6T 1Z4, Canada; 2 Centre de Recherche en Infectiologie de l'Université Laval, Centre Hospitalier Universitaire de Québec (Pavilion CHUL), Quebec City, QC, G1V 4G2, Canada; 3 Groupe de Recherche en Écologie Buccale, Faculté de Médecine Dentaire, Université Laval, Quebec City, QC, G1V 0A6, Canada

## Abstract

**Background:**

Streptococci are divided into six phylogenetic groups, *i.e*, anginosus, bovis, mitis, mutans, pyogenic, and salivarius, with the salivarius group consisting of only three distinct species. Two of these species, *Streptococcus salivarius *and *Streptococcus vestibularis*, are members of the normal human oral microflora whereas the third, *Streptococcus thermophilus*, is found in bovine milk. Given that *S. salivarius *and *S. vestibularis *share several physiological characteristics, in addition to inhabiting the same ecosystem, one would assume that they would be more closely related to each other than to *S. thermophilus*. However, the few phylogenetic trees published so far suggest that *S. vestibularis *is more closely related to *S. thermophilus*. To determine whether this phylogenetic relationship is genuine, we performed phylogenetic inferences derived from *secA *and *secY*, the general secretion housekeeping genes, *recA*, a gene from a separate genetic locus that encodes a major component of the homologous recombinational apparatus, and 16S rRNA-encoding gene sequences using other streptococcal species as outgroups.

**Results:**

The maximum likelihood (ML) and maximum parsimony (MP) phylogenetic inferences derived from the *secA *and *recA *gene sequences provided strong support for the *S. vestibularis*/*S. thermophilus *sister-relationship, whereas 16S rRNA-encoding and *secY*-based analyses could not discriminate between alternate topologies. Phylogenetic analyses derived from the concatenation of these sequences unambiguously supported the close affiliation of *S. vestibularis *and *S. thermophilus*.

**Conclusion:**

Our results corroborated the sister-relationship between *S. vestibularis *and *S. thermophilus *and the concomitant early divergence of *S. salivarius *at the base of the salivarius lineage.

## Background

The *Streptococcus *genus comprises ninety-two recognized species that are present in a wide variety of habitats [1]. In humans and animals, a number of streptococcal species are important pathogens (*e.g*., *S. pneumoniae*, *S. pyogenes, S. suis*, and *S. mutans*), while others are members of mutualistic microflora (*e.g*., *S. oralis*, *S. downei*, *S. dentirousetti*, and *S. salivarius*). The species of the *Streptococcus *genus have been divided into six groups (anginosus, bovis, mitis, mutans, pyogenic, and salivarius) based on 16S rDNA phylogenetic inferences [2]. According to these authors, the salivarius group is composed of three species: (1) *S. salivarius*, a pioneer colonizer of the human oral mucosa that is isolated mainly from the dorsum of the tongue, the cheeks, and the palate [3], (2) *S. vestibularis*, a mutualistic bacterium that is present on the vestibulum of the human oral mucosa [4], and (3) *S. thermophilus*, a thermophilic species [5] that is part of starter cultures used in the production of yogurt and Swiss- or Italian-type cooked cheeses. Unlike *S. salivarius *and *S. vestibularis*, *S. thermophilus *is not a natural inhabitant of the human oral mucosa and is commonly found on the mammary mucosa of bovines, its natural ecosystem, as inferred from its presence and that of *thermophilus*-specific bacteriophages in raw milk isolates [6-8].

The common ecosystem is not the only feature shared by *S. salivarius *and *S. vestibularis*. Biochemical investigations of functional metabolic pathways have revealed that these two species share a high level of physiological resemblance. For example, *S. salivarius *and *S. vestibularis *are capable of hydrolyzing esculin and generating acidic compounds from maltose and *N*-acetyl-glucosamine, while *S. thermophilus *is not ([9] and references therein). Both *S. salivarius *and *S. vestibularis *are also opportunistic pathogens that can cause mild to severe infective endocarditis [10-12], whereas *S. thermophilus *has never been implicated in such infections. Given the home environments of the organisms, the high level of metabolic similarity between *S. salivarius *and *S. vestibularis*, and the more restricted spectrum of carbon sources that can be used by *S. thermophilus *[13], one would assume that *S. salivarius *and *S. vestibularis *would be more related to each other than to *S. thermophilus*. However the few phylogenetic trees published so far that include all three species, as inferred from 16S rRNA-encoding gene sequences [2] and the housekeeping gene *sodA *that encodes the manganese-dependent superoxide dismutase [14], suggest that a schism generated *S. vestibularis *and *S. thermophilus *subsequent to the early divergence of *S. salivarius*. However, since these two phylogenetic studies [2,14] were limited to only one taxon for each species, the inferred relationships between these three species might be inaccurate.

To investigate the evolutionary relationships between the three species making up the salivarius group, we performed phylogenetic inferences based on the 16S rRNA-encoding, *secA *and *secY *housekeeping genes and the important yet non-essential *recA *gene using an identical distribution of streptococcal strains among the various markers to facilitate direct comparisons and allow the concatenation of the individual sequences into a single matrix. These four ubiquitous genes are widely distributed and have homologues in all three kingdoms, *i.e*., *Bacteria, Archaea*, and *Eukarya *(for reviews see [15-17]). The 16S rRNA-encoding gene, which codes for the major ribonucleic constituent of the bacterial small ribosomal subunit [18], is one the most frequently used housekeeping phylogenetic markers [19], while the *secA *and *secY *genes code for components of the general protein-secretion pathway, which is essential for several cell functions [20]. The fourth gene, *recA*, codes for a product that initiates the formation of Holliday junction intermediates during homologous recombination [21]. Our ML and MP phylogenetic inferences based of these four gene sequences are in agreement with earlier findings by Kawamura *et al*. [2] and Poyart *et al*. [14] and corroborate the *S. thermophilus*/*S. vestibularis *sister-relationship.

## Results

### Phylogenetic analyses of *secA *gene sequences

**Figure 1:**
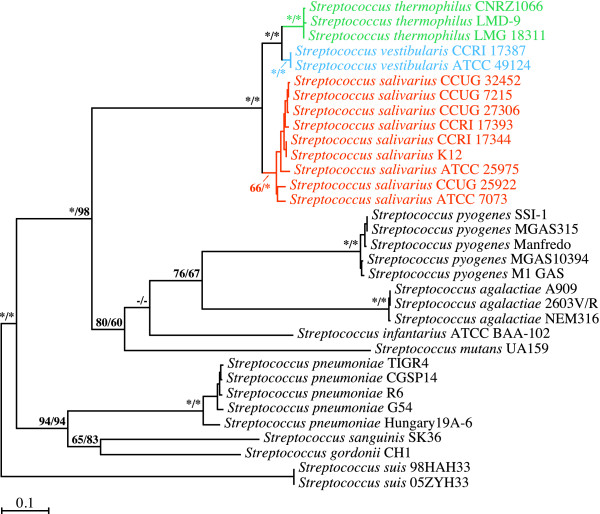
**Branching order of members of the salivarius group as inferred from ML and MP analyses of *secA *gene sequences (2484 positions; 1261 variable, 1169 phylogenetically informative)**. The best ML tree computed with PHYML 3.0 under the GTR+Γ4+I model of nucleotide substitution is shown here. Bootstrap support for the major nodes is indicated over the corresponding nodes: ML values left, MP values right. Asterisks denote nodes that were retrieved in all the bootstrap replicates. Dashes indicate nodes that were retrieved in fewer than 50% of the bootstrap replicates. Streptococcal species belonging to the salivarius group are shown in orange (*S. salivarius*), blue (*S. vestibularis*), or green (*S. thermophilus*). Strains CCUG 7215 and CCUG 27306, which are categorized as *Streptococcus vestibularis *in the CCUG culture collection, are capable of using raffinose as the sole carbon source. This contradicts Whiley and Hardie's [4] canonical *S. vestibularis *species definition. This metabolic trait is more a hallmark of the closely related *Streptococcus salivarius *species, to which the two strains belong. Other streptococcal species shown in black were outgroups. Branch lengths are drawn to scale.

We began our investigation of the branching order of the streptococci of the salivarius group by looking at phylogenetic trees inferred from the *secA *gene (Figure 1). As expected, the salivarius group comprising *S. salivarius*, *S. thermophilus*, and *S. vestibularis *was monophyletic in all the ML and MP bootstrap replicates. The *S. thermophilus *and *S. vestibularis *species monophylies were strongly supported by the ML and MP analyses, while support for the *S. salivarius *monophyly ranged from weak to moderate in the ML analyses and strong in the MP analyses. Our phylogenetic analyses based on *secA *gene sequences strongly support the notion that *S. vestibularis *and *S. thermophilus *are closely related species. The node comprising these two species was retrieved in all the ML and MP bootstrap replicates, while the other two possible alternate topologies, *i.e*., the *S. salivarius*/*S. vestibularis *and *S. salivarius*/*S. thermophilus *relationships, were not recovered in any of the replicates.

### Phylogenetic analyses of *secY *gene sequences

**Figure 2:**
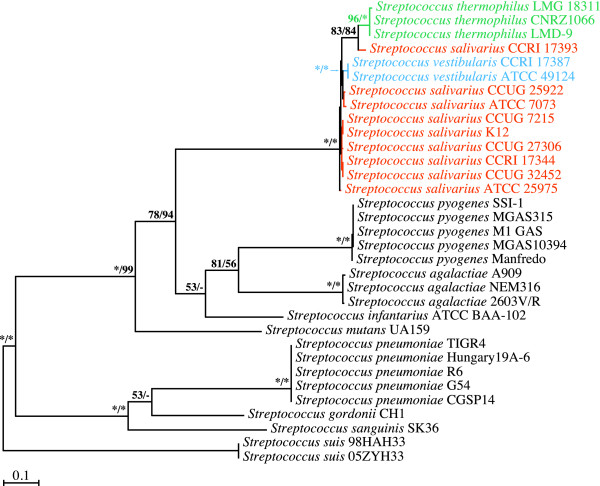
**Branching order of members of the salivarius group as inferred from ML and MP analyses of *secY *gene sequences (1287 positions; 735 variable, 686 phylogenetically informative)**. The best ML tree computed with PHYML 3.0 under the GTR+Γ4+I model of nucleotide substitution is shown here. Bootstrap support for the major nodes is indicated over the corresponding nodes: ML values left, MP values right. Asterisks denote nodes that were retrieved in all the bootstrap replicates. Dashes indicate nodes that were retrieved in fewer than 50% of the bootstrap replicates. Streptococcal species belonging to the salivarius group are shown in orange (*S. salivarius*), blue (*S. vestibularis*) or green (*S. thermophilus*). Other streptococcal species shown in black were outgroups. Branch lengths are drawn to scale.

The ML and MP phylogenetic inferences derived from the *secY *gene were not as conclusive (Figure 2). Although the monophyly of the salivarius group was again recovered in all the bootstrap replicates, together with the unambiguous delineation of the *S. vestibularis *and *S. thermophilus *species, the *S. salivarius *species was paraphyletic, with *S. salivarius *strain CCRI 17393 branching out at the base of the three *S. thermophilus *strains. However, given the differences in branch lengths between *S. salivarius *strain CCRI 17393 and the other *S. salivarius *strains, the positioning of this strain at the base of the *S. thermophilus *strains appears dubious and may result from artifactual attraction between locally long branches, an effect that might have been exacerbated by the scarcity of informative characters in this dataset. Of the 1287 positions constituting the *secY *dataset, 135 displayed variations between members of the salivarius group, with only 98 being phylogenetically informative (Table 1). In contrast, the *secA *dataset featured 266 variable sites, with 222 phylogenetically informative characters among members of the salivarius group, *i.e*., more than twice the amount of potentially discriminating information. On the other hand, we cannot exclude the possibility that the branching of *S. salivarius *strain CCRI 17393 at the base of the *S. thermophilus *strains in our *secY*-based analyses resulted from a genuine phylogenetic signal. If this is true, then the *secA *and *secY *gene sequences from *S. salivarius *strain CCRI 17393 have evolved in different directions. In any event, the phylogenetic resolution of the *secY *dataset was not sufficient to unambiguously infer the branching order between the three species making up the salivarius group.

**Table 1:** Main features of each phylogenetic dataset

		Full Dataset		**Salivarius Subset**^**c**^	
		
Name	Length	**Variable**^**a**^	**Informative**^**b**^	**Variable**^**a**^	**Informative**^**b**^
*secA*	2484	1261	1169	266	222
*secY*	1287	735	686	135	98
*recA*	798	309	289	102	96
16S	1374	169	141	14	8
All^d^	5943	2474	2285	517	424

^a ^Number of variable characters

^b ^Number of phylogenetically informative characters

^c ^Values observed between the 14 *S. salivarius*, *S. thermophilus*, and *S. vestibularis *taxa

^d ^Dataset containing the 16S rRNA-encoding, *recA*, *secA*, and *secY *concatenated gene sequences

### Phylogenetic analyses of *recA *partial gene sequences

**Figure 3:**
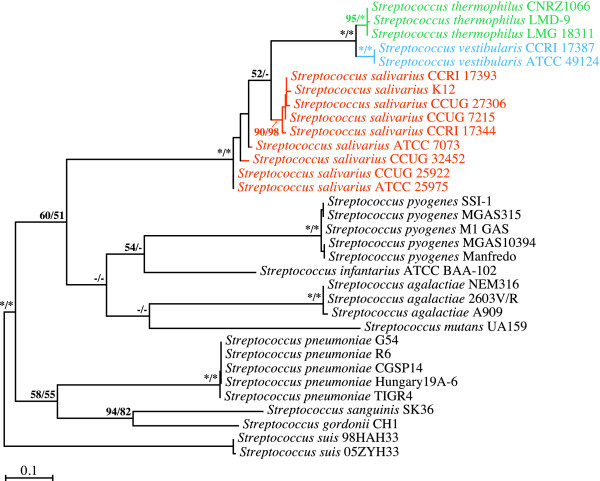
**Branching order of members of the salivarius group as inferred from ML and MP analyses of *recA *partial gene sequences (798 positions; 309 variable, 289 phylogenetically informative)**. The best ML tree computed with PHYML 3.0 under the GTR+Γ4+I model of nucleotide substitution is shown here. Bootstrap support for the major nodes is indicated over the corresponding nodes: ML values left, MP values right. Asterisks denote nodes that were retrieved in all the bootstrap replicates. Dashes indicate nodes that were retrieved in fewer than 50% of the bootstrap replicates. Streptococcal species belonging to the salivarius group are shown in orange (*S. salivarius*), blue (*S. vestibularis*) or green (*S. thermophilus*). Other streptococcal species shown in black were outgroups. Branch lengths are drawn to scale.

Our phylogenetic inferences based on *recA *partial gene sequences yielded clearer insights into the branching order of the members of the salivarius group (Figure 3), which were clustered together in all the ML and MP bootstrap replicates, while the two *S. vestibularis *strains formed a united clade in all the replicates, and the three *S. thermophilus *strains branched together in the vast majority of the bootstrap replicates. The monophyly of the *S. salivarius *species was recovered in 98% of the MP bootstrap replicates, although ML-based phylogenetic inferences could not discriminate between paraphyletic and monophyletic *S. salivarius *clades (52% vs. 48% of the bootstrap replicates, respectively). Like the *secA*-based phylogenetic inferences, the analyses derived from the *recA *gene sequences strongly supported a sister-relationship between the *S. vestibularis *and *S. thermophilus *species. The node comprising these two species was robust and was recovered in all the ML and MP bootstrap replicates.

### Phylogenetic analyses of 16S rRNA-encoding gene sequences

**Figure 4:**
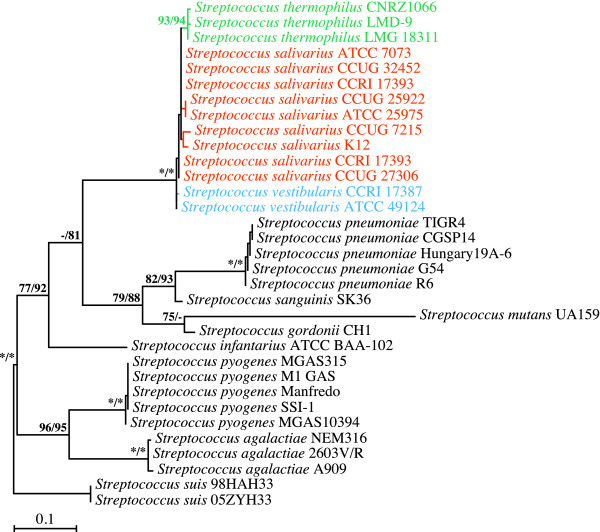
**Branching order of members of the salivarius group as inferred from ML and MP analyses of 16S rRNA-encoding partial gene sequences (1374 positions; 169 variable, 141 phylogenetically informative)**. The best ML tree computed with PHYML 3.0 under the GTR+Γ4+I model of nucleotide substitution is shown here. Bootstrap support for the major nodes is indicated over the corresponding nodes: ML values left, MP values right. Asterisks denote nodes that were retrieved in all the bootstrap replicates. Dashes indicate nodes that were retrieved in fewer than 50% of the bootstrap replicates. Streptococcal species belonging to the salivarius group are shown in orange (*S. salivarius*), blue (*S. vestibularis*), or green (*S. thermophilus*). Other streptococcal species shown in black were outgroups. Branch lengths are drawn to scale.

Building on the phylogeny published by Kawamura *et al*. [2], we reinvestigated the branching order among the salivarius streptococci using 16S rRNA-encoding gene sequences and expanded taxon sampling within the salivarius group. As can be seen in Figure 4, even though the salivarius group was recovered in all the bootstrap replicates, the branching order within this taxonomic entity was not well defined. Of the three species, only *S. thermophilus *composed a monophyletic assemblage. The other two, *S. vestibularis *and *S. salivarius*, were not resolved. This contrasted with the results obtained by Kawamura *et al*. [2], who reported that the *S. vestibularis *and *S. thermophilus *species branched together with strong bootstrap support. It should be noted, however, that the 16S rRNA-encoding gene sequences exhibited almost no variability among salivarius streptococci. Of the 1374 positions making up our 16S rRNA-encoding gene dataset, only 14 were variable, with a mere eight positions giving tangible phylogenetic information for the three salivarius species (Table 1). The topologies inferred from the 16S rRNA-encoding gene sequences should thus be treated with caution with respect to the branching order of salivarius streptococci.

### Phylogenetic analyses of concatenated gene sequences

**Figure 5:**
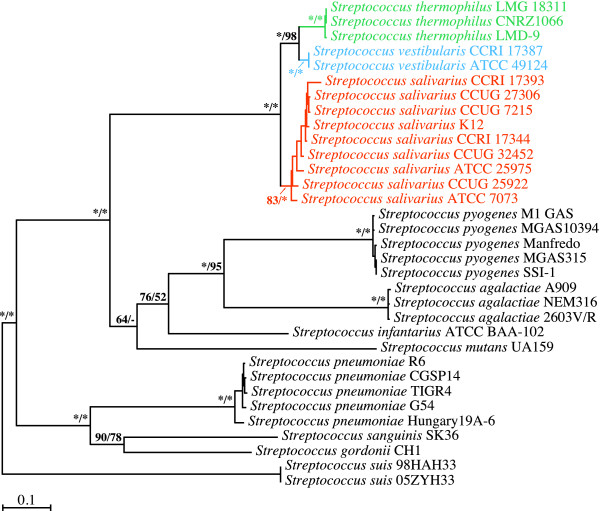
**Branching order of members of the salivarius group as inferred from ML and MP analyses of concatenated 16S rRNA-encoding, *recA*, *secA*, and *secY *gene sequences (5943 positions; 2474 variable, 2285 phylogenetically informative)**. The best ML tree computed with PHYML 3.0 under the GTR+Γ4+I model of nucleotide substitution is shown here. Bootstrap support for the major nodes is indicated over the corresponding nodes: ML values left, MP values right. Asterisks denote nodes that were retrieved in all the bootstrap replicates. Dashes indicate nodes that were retrieved in fewer than 50% of the bootstrap replicates. Streptococcal species belonging to the salivarius group are shown in orange (*S. salivarius*), blue (*S. vestibularis*) or green (*S. thermophilus*). Other streptococcal species shown in black were outgroups. Branch lengths are drawn to scale.

To increase the resolving power of our phylogenetic analyses, we concatenated the four previous datasets into a single matrix to pool their phylogenetic signals. As anticipated, our ML and MP analyses based on the concatenated *secA*, *secY*, *recA*, and 16S rRNA-encoding gene sequences yielded superior resolved topologies (Figure 5). While the clade constituting the salivarius group and the monophylies of the *S. thermophilus *and *S. vestibularis *species were once again recovered in all of the bootstrap replicates, support for the monophyly of the *S. salivarius *species increased appreciably. In the ML analyses, the concatenation of the various datasets had a synergistic effect on the *S. salivarius *monophyly for which bootstrap support attained a level not seen with any of the independent gene datasets. In the MP analyses, the bootstrap support for this monophyly remained strong. The phylogenetic inferences derived from the concatenated *secA*, *secY*, *recA*, and 16S rRNA-encoding gene sequences strongly supported the sister-relationship between the *S. vestibularis *and *S. thermophilus *species. This sister-relationship and the concomitant early divergence of the *S. salivarius *species at the base of the salivarius clade were recovered in 100% and 98% of the ML and MP bootstrap replicates, respectively.

## Discussion

When we began our study, we expected that the *S. salivarius *and *S. vestibularis *species would be more closely related to each other given their level of physiological resemblance and that the *S. vestibularis*/*S. thermophilus *sister-relationship inferred in previous phylogenetic studies [2,14] would not be robustly supported. Obviously, this was not the case. Our results were in complete agreement with earlier neighbor-joining phylogenies based on partial 16S rRNA-encoding and *sodA *gene sequences [2,14] and corroborated the *S. vestibularis*/*S. thermophilus *sister-relationship. This sister-relationship was not dependent on the method of phylogenetic reconstruction and was strongly supported by both our ML and MP analyses. Furthermore, while the 16S-rRNA-encoding and *secY *gene sequences were unable to discriminate between the *S. vestibularis*/*S. thermophilus *and the alternate *S. vestibularis*/*S. salivarius *and *S. salivarius*/*S. thermophilus *sister-relationships, we observed no serious incongruities between the topologies inferred from these molecular markers and those inferred from the *recA *and *secA *gene sequences.

The *S. vestibularis*/*S. thermophilus *sister-relationship inferred from our phylogenetic analyses is not necessarily incompatible with the observation that *S. vestibularis *share more phenotypic similarities with *S. salivarius *than with *S. thermophilus*. Following speciation from a putative common ancestor physiologically similar to *S. salivarius*, the two newly formed species could have evolved differently, with *S. vestibularis *and *S. thermophilus *independently retaining and discarding a number of ancestral features. Many of the phenotypic losses observed in the *S. thermophilus *species could have been induced by its adaptation to its new ecosystem, *i.e*., the bovine mammary mucosa. In particular, because this species has access to a wealth of nutrients within bovine milk, polyvalence for sugar metabolism-related genes might not be as important for this species as for its relatives inhabiting the human oral mucosa [13]. Further losses could have been caused by additional selective pressure applied on *S. thermophilus *commercial strains ([22] and references therein) that are used in the manufacture of various dairy products.

The relationships inferred among the three salivarius streptococci raise interesting questions regarding their establishment in their respective ecosystems. Because the *S. salivarius/S. vestibularis *sister-relationship is not supported by phylogenetic analyses, the colonization of the human oral cavity by an ancestor of *S. thermophilus *present in bovine milk, which would have then speciated over time into *S. salivarius *and *S. vestibularis*, is not plausible. Furthermore, the independent colonization of bovine mammary and human oral mucosae by a putative ancestor originating from a third environment is not compatible with these phylogenies unless we assume two distinct yet closely related streptococcal ancestors; one that independently colonized the two ecosystems yielding *S. thermophilus *and *S. vestibularis *on the one hand, and *S. salivarius *on the other. Alternatively, the direct or indirect invasion of the bovine mammary mucosa by an ancestor of *S. vestibularis *originating from the human oral cavity would also be compatible with the *S. vestibularis*/*S. thermophilus *sister-relationship.

## Conclusion

The phylogenetic analyses presented in the present paper strongly support the *S. vestibularis*/*S. thermophilus *sister-relationship and the concomitant early divergence of *S. salivarius *at the base of the salivarius clade, which is in agreement with previous 16S rDNA/*sodA*-based phylogenetic inferences [2,14]. One of the main reasons for conducting the present study was the paucity of phylogenetic studies involving all three species making up the salivarius group. Although a number of studies that included *S. salivarius *and *S. vestibularis *have been published, *S. thermophilus *has been omitted more often than not since it is not retrieved from human clinical isolates. Since the complete genome sequences of three *S. thermophilus *strains are now available, it would be interesting to revisit phylogenetic studies that involve different phylogenetic markers and *S. salivarius/S. vestibularis *but not *S. thermophilus *to verify whether the addition of *S. thermophilus *would result in a similar branching order among salivarius streptococci.

## Methods

### Source organisms

*Streptococcus salivarius *strains ATCC 7073 and 25975 and *Streptococcus vestibularis *strain ATCC 49124 were obtained from the American Type Culture Collection (Manassas, VA, USA). *Streptococcus salivarius *strain K12 was obtained from BLIS Technologies Ltd. (Dunedin, New Zealand). *Streptococcus salivarius *strains CCUG 32452 and 25922 and *Streptococcus vestibularis *strains CCUG 7215 and 27306 (renamed *S. salivarius *strains CCUG 7215 and 27306 herein) were obtained from the University of Göteborg Culture Collection (Göteborg, Sweden). *Streptococcus salivarius *clinical isolates CCRI 17344 and CCRI 17393 and *Streptococcus vestibularis *clinical isolate CCRI 17387 were obtained from the Centre de Recherche en Infectiologie of the Centre Hospitalier Universitaire de Québec (CHUQ), CHUL Pavilion (Quebec City, QC, Canada). The identity of the *S. vestibularis *strains was confirmed by comparative growth on TYE medium containing either raffinose or glucose as the sole carbon source.

### DNA isolation and sequencing

Streptococcal strains were grown in TYE-glucose liquid medium as described in Lévesque *et al*. [23] or on sheep-blood agar medium overnight at 35°C in a 5% CO_2 _atmosphere. Their 16S rRNA-encoding, *recA*, *secA*, and *secY *genes were amplified by polymerase chain reaction (PCR) from either (A) purified chromosomal DNA, (B) DNA released from boiled bacterial colonies, or (C) bacterial lysates. Purified chromosomal DNA was obtained as follows. Streptococcal cells were pelleted by centrifugation. The pellets were washed for 30 min at 37°C in 50 mM Tris-HCl buffer (pH 8) containing 6.7% (w/v) sucrose, 1 mM EDTA, and 40 U/ml of mutanolysin. SDS (final concentration 1%) was then added and the cells were lysed for 10 min at 60°C. Proteinase K (final concentration 0.14 mg/ml) was added and the incubation was continued for an additional 20 min. Chromosomal DNA was isolated from the cellular debris using the standard phenol/ChCl_3 _extraction protocol described by Sambrook *et al*. [24]. DNA released from boiled cells was obtained as follows. Streptococcal colonies grown on TYE-glucose agar or blood agar medium were suspended in 100 μl of distilled water and then boiled at 94°C for 3 min. This suspension was then used instead of sterile distilled water in the PCR protocols. Bacterial lysates were obtained with the BD GeneOhm™ Lysis Kit (BD Diagnostics-GeneOhm, Quebec City, QC, Canada). The 16S rRNA-encoding, *recA, secA *and *secY *genes were amplified by PCR using primers 16S_F (5'-AGTTTGATCCTGGCTCAGGACG-3') and 16S_R (5'-ATCCAGCCGCACCTTCCGATAC-3'), SSU27 (5'-AGAGTTTGATCMTGGCTCAG-3') and SSU1492 (5'-TACGGYTACCTTGTTACGACTT-3'), RStrGseq81 (5'-GAAAWWIATYGARAAAGAITTTGGTAA-3') and RStrGseq937 (5'-TTYTCAGAWCCTTGICCAATYTTYTC-3'), SecAAMON (5'-CAGGCCTTTGAAAATCTCTTAC-3') and SecAAVAL (5'-CTCTTTATCACGAGCTTGCTTC-3'), or SecYAMON (5'-CTGCTGAAGCAGCTATCACTGC-3') and SecYAVAL (5'-CTTTACCAGCACCTGGTAGACC-3'). The PCR templates were sequenced using Sanger dideoxynucleotide chemistry as described in Pombert *et al*. [25]. The sequences were edited and assembled using STADEN package version 1.7.0 http://staden.sourceforge.net/ or SEQUENCHER 4.8 (GeneCodes, Ann Arbor, MI, USA).

### Dataset preparation

The sequences we used were either retrieved from GenBank or sequenced by the authors. Sequences showing ambiguous base calling in databases were not selected for phylogenetic analyses. The 16S rRNA-encoding gene sequences were aligned using CLUSTALX 2.0.7 [26], whereas the *recA*, *secA*, and *secY *gene sequences were aligned by positioning their codons on the corresponding protein alignments. To do so, the amino acid sequences from the corresponding gene sequences were first deduced using the bacterial translation table from GETORF in EMBOSS 6.0.1 [27]. They were then aligned using CLUSTALX 2.0.7, and the codons were positioned according to the amino acid alignments. Ambiguous regions in the alignments were filtered out with GBLOCKS 0.91b [28]. A fifth dataset was produced by concatenating the resulting filtered sequences. Bootstrap replicates for the ML analyses were generated with SEQBOOT from the PHYLIP 3.67 package [29].

### Phylogenetic analyses

Computations and analyses were performed on a cluster of four AMD64 Opteron 875 Dual Core 2.2 Ghz/2 MB L2 cache CPUs with 16 GB of RAM running on Debian 4.0 (kernel 2.6.16.21), a MacBook Pro laptop with a Core 2 Duo 2.4 Ghz/4 MB L2 cache CPU and 2 GB of RAM running on MacOS X 10.5.4, or on an ASUS M6NBe laptop with a 1.6 G Hz/2 MB L2 cache Dothan CPU and 1 GB of RAM running on MS Windows XP SP3. Maximum likelihood (ML) analyses were computed using PHYML 3.0 [30] under the GTR + Γ4 +I nucleotide substitution model. This model was selected using the Akaike Information Criterion (Akaike 1973), as implemented using jModelTest 3.7 [31]. One hundred bootstrap replicates were performed for each ML analysis. Maximum parsimony (MP) analyses were performed with PAUP* 4.0b10 [32], each using a thousand bootstrap replicates.

### Accession numbers

Nucleotide sequence data reported are available in the GenBank database under accession numbers [GenBank: FJ154797] to [GenBank: FJ154838] (Table 2).

**Table 2:** Accession numbers

	Accession Numbers			
	
Streptococci	*recA*	*secA*	*secY*	16S rDNA
*S. agalactiae* 2603V/R	[GenBank: NC_004116]	[GenBank: NC_004116]	[GenBank: NC_004116]	[GenBank: NC_004116]
*S. agalactiae* A909	[GenBank: NC_007432]	[GenBank: NC_007432]	[GenBank: NC_007432]	[GenBank: NC_007432]
*S. agalactiae* NEM316	[GenBank: NC_004368]	[GenBank: NC_004368]	[GenBank: NC_004368]	[GenBank: NC_004368]
*S. gordonii *str. Challis substr. CH1	[GenBank: NC_009785]	[GenBank: NC_009785]	[GenBank: NC_009785]	[GenBank: NC_009785]
*S. infantarius* ATCC BAA-102	[GenBank: NZ_ABJK02000015]	[GenBank: NZ_ABJK02000019]	[GenBank: NZ_ABJK02000013]	[GenBank: AF429762]
*S. mutans* UA159	[GenBank: NC_004350]	[GenBank: NC_004350]	[GenBank: NC_004350]	[GenBank: NC_004350]
*S. pneumoniae* CGSP14	[GenBank: NC_010582]	[GenBank: NC_010582]	[GenBank: NC_010582]	[GenBank: NC_010582]
*S. pneumoniae* G54	[GenBank: NC_011072]	[GenBank: NC_011072]	[GenBank: NC_011072]	[GenBank: NC_011072]
*S. pneumoniae* Hungary19A-6	[GenBank: NC_010380]	[GenBank: NC_010380]	[GenBank: NC_010380]	[GenBank: NC_010380]
*S. pneumoniae* R6	[GenBank: NC_003098]	[GenBank: NC_003098]	[GenBank: NC_003098]	[GenBank: NC_003098]
*S. pneumoniae* TIGR4	[GenBank: NC_003028]	[GenBank: NC_003028]	[GenBank: NC_003028]	[GenBank: NC_003028]
*S. pyogenes *M1 GAS	[GenBank: NC_002737]	[GenBank: NC_002737]	[GenBank: NC_002737]	[GenBank: NC_002737]
*S. pyogenes* MGAS10394	[GenBank: NC_006086]	[GenBank: NC_006086]	[GenBank: NC_006086]	[GenBank: NC_006086]
*S. pyogenes* MGAS315	[GenBank: NC_004070]	[GenBank: NC_004070]	[GenBank: NC_004070]	[GenBank: NC_004070]
*S. pyogenes* SSI-1	[GenBank: NC_004606]	[GenBank: NC_004606]	[GenBank: NC_004606]	[GenBank: NC_004606]
*S. pyogenes *str. Manfredo	[GenBank: NC_009332]	[GenBank: NC_009332]	[GenBank: NC_009332]	[GenBank: NC_009332]
*S. salivarius* ATCC 25975	[GenBank: FJ154806^b^]	[GenBank: FJ154817^b^]	[GenBank: FJ154828^b^]	[GenBank: FJ154797^b^]
*S. salivarius* ATCC 7073	[GenBank: FJ154807^b^]	[GenBank: FJ154818^b^]	[GenBank: FJ154829^b^]	[GenBank: AY188352]
*S. salivarius* CCRI 17344	[GenBank: FJ154808^b^]	[GenBank: FJ154819^b^]	[GenBank: FJ154830^b^]	[GenBank: FJ154798^b^]
*S. salivarius* CCRI 17393	[GenBank: FJ154809^b^]	[GenBank: FJ154820^b^]	[GenBank: FJ154831^b^]	[GenBank: FJ154799^b^]
*S. salivarius* CCUG 25922	[GenBank: FJ154810^b^]	[GenBank: FJ154821^b^]	[GenBank: FJ154832^b^]	[GenBank: FJ154800^b^]
*S. salivarius* CCUG 27306^a^	[GenBank: FJ154811^b^]	[GenBank: FJ154822^b^]	[GenBank: FJ154833^b^]	[GenBank: FJ154801^b^]
*S. salivarius* CCUG 32452	[GenBank: FJ154812^b^]	[GenBank: FJ154823^b^]	[GenBank: FJ154834^b^]	[GenBank: FJ154802^b^]
*S. salivarius* CCUG 7215^a^	[GenBank: FJ154813^b^]	[GenBank: FJ154824^b^]	[GenBank: FJ154835^b^]	[GenBank: FJ154803^b^]
*S. salivarius* K12	[GenBank: FJ154814^b^]	[GenBank: FJ154825^b^]	[GenBank: FJ154836^b^]	[GenBank: FJ154804^b^]
*S. sanguinis* SK36	[GenBank: NC_009009]	[GenBank: NC_009009]	[GenBank: NC_009009]	[GenBank: NC_009009]
*S. suis* 05ZYH33	[GenBank: NC_009442]	[GenBank: NC_009442]	[GenBank: NC_009442]	[GenBank: NC_009442]
*S. suis* 98HAH33	[GenBank: NC_009443]	[GenBank: NC_009443]	[GenBank: NC_009443]	[GenBank: NC_009443]
*S. thermophilus* CNRZ1066	[GenBank: NC_006449]	[GenBank: NC_006449]	[GenBank: NC_006449]	[GenBank: NC_006449]
*S. thermophilus* LMD-9	[GenBank: NC_008532]	[GenBank: NC_008532]	[GenBank: NC_008532]	[GenBank: NC_008532]
*S. thermophilus* LMG 18311	[GenBank: NC_006448]	[GenBank: NC_006448]	[GenBank: NC_006448]	[GenBank: NC_006448]
*S. vestibularis* ATCC 49124	[GenBank: FJ154815^b^]	[GenBank: FJ154826^b^]	[GenBank: FJ154837^b^]	[GenBank: AY188353]
*S. vestibularis* CCRI 17387	[GenBank: FJ154816^b^]	[GenBank: FJ154827^b^]	[GenBank: FJ154838^b^]	[GenBank: FJ154805^b^]

^a ^Erroneously categorized as *Streptococcus vestibularis *in the culture collection of the University of Göteborg (CCUG)

^b ^This study

## Authors' contributions

JFP designed the study, verified the phenotypic validation of the *S. vestibularis *strains, sequenced the 16S RNA-encoding, *secA*, and *secY *genes with the help of VS, prepared the accession numbers, performed the data mining, sequence alignments and phylogenetic analyses, generated the figures and tables, and drafted the manuscript. VS participated in the 16S RNA-encoding, *secA*, and *secY *gene sequencing and determined the *recA *gene sequences. MB coordinated the work of VS and the isolation of CCRI streptococcal strains. MF participated in the design and coordination of the study and helped draft the manuscript. All the authors have read and approved the final manuscript.
